# Aortic Dissection due to Eosinophilic Granulomatosis With Polyangiitis

**DOI:** 10.7759/cureus.10167

**Published:** 2020-08-31

**Authors:** Hiroshi Ito

**Affiliations:** 1 Division of Hospital Medicine, University of Tsukuba Hospital, Tsukuba, JPN

**Keywords:** aortic dissection, eosinophilic granulomatosis with polyangiitis

## Abstract

Eosinophilic granulomatosis with polyangiitis (EGPA) usually presents as a systemic vasculitis of small- and middle-sized blood vessels. There has been no report of this disorder accompanying aortic involvement. A 61-year-old woman with EGPA was admitted to the hospital because of type B aortic dissection. She was treated medically, and the lesion did not worsen during her hospitalization. EGPA can present as aortic involvement, and conservative treatment is useful in managing this condition. Clinicians should be aware of the possibility of aortic involvement in patients with EGPA who have sudden back pain.

## Introduction

Eosinophilic granulomatosis with polyangiitis (EGPA) is a systematic vasculitis of the small vessels and is characterized by the expression of myeloperoxidase antineutrophil cytoplasmic antibody (MPO-ANCA). EGPA was defined in the 2012 International Chapel Hill Consensus Conference as a necrotizing vasculitis affecting small- to medium-sized vessels and is associated with asthma and eosinophilia [1]. It is believed that large vessels such as the aorta are rarely affected by EGPA. To the best of my knowledge, this is the first report of a patient with EGPA who developed aortic dissection, which was successfully managed by conservative treatment.

## Case presentation

**Figure 1:**
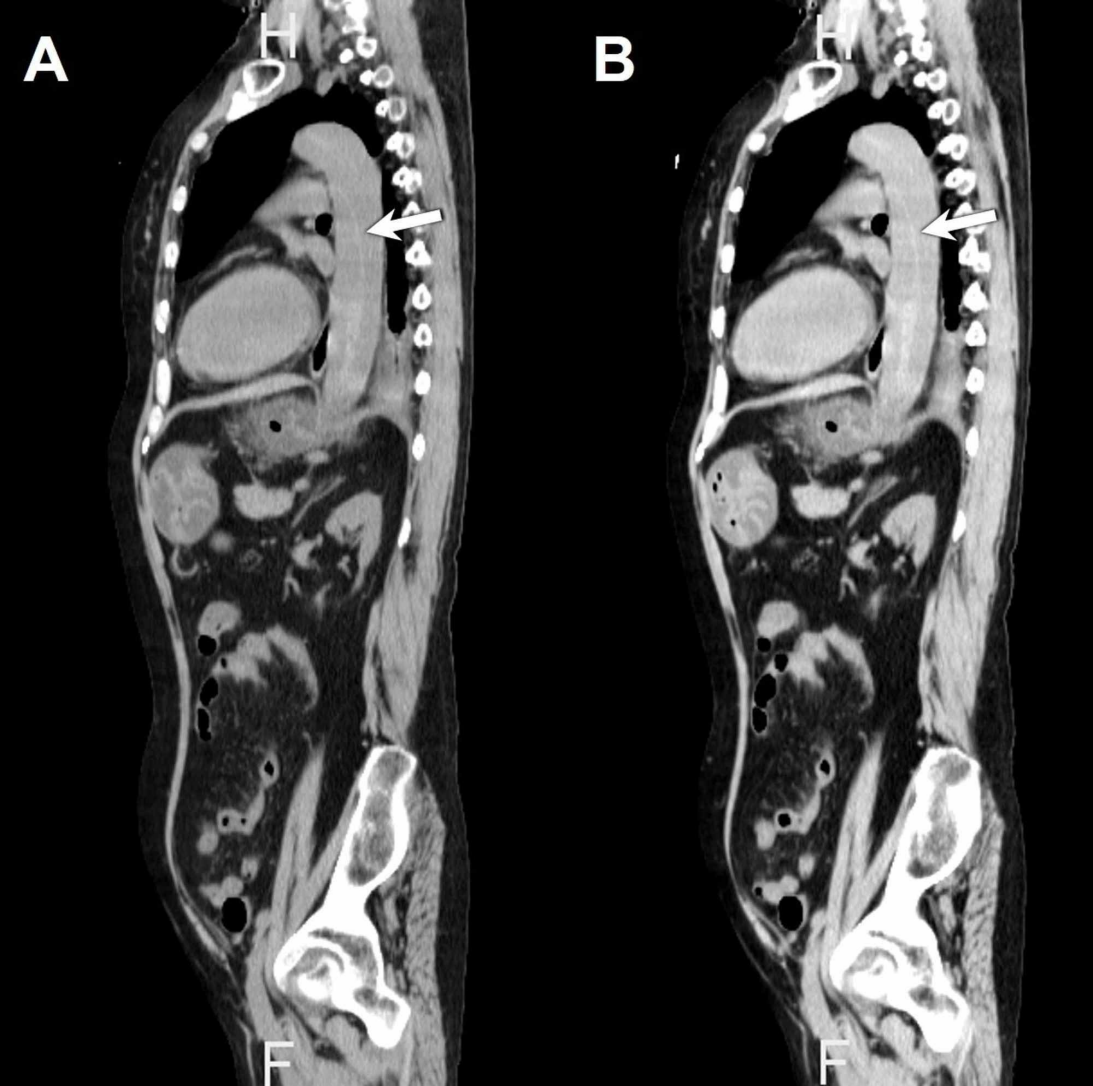
Precontrast CT scans of the aorta (A) The descending aorta was dissected (arrow). (B) The aortic dissection did not worsen during the hospitalization (arrow).

A 61-year-old Japanese woman was admitted to our hospital after the sudden onset of severe back pain. She had been diagnosed with EGPA seven years before, based on bronchial asthma, rapidly progressive glomerulonephritis, peripheral neuropathy, eosinophilia, and positive MPO-ANCA. The EGPA had been in remission following treatment with 10 mg of oral prednisolone daily. She had been known to have hypertension because of EGPA, which was under control with antihypertensives. Her medical history was otherwise unremarkable. On admission, her blood pressure was 199/107 mmHg, with a regular pulse rate of 87 bpm. Laboratory data were as follows: white blood cell count 24,400/μL, hemoglobin 11.9 g/dL, platelet count 183,000/μL, serum creatinine 1.82 mg/dL, and C-reactive protein 0.25 mg/dL. Precontrast CT scans revealed dissection of the descending aorta (Figure 1A). She was diagnosed with type B aortic dissection, and intravenous nicardipine and bisoprolol transdermal patches were administered. Her systolic blood pressure was kept below 120 mmHg, and her heartbeat was kept below 70 bpm [2]. She also started to take 15 mg of oral prednisolone daily as a steroid cover. She undertook rehabilitation, and her aortic dissection did not worsen during her hospitalization (Figure 1B). She was discharged on the 24th hospital day and prescribed oral nifedipine, losartan, carvedilol, and 12.5 mg of prednisolone daily.

## Discussion

EGPA, formerly known as Churg-Strauss syndrome, is a necrotizing vasculitis predominantly affecting small- to medium-sized vessels and is associated with asthma and eosinophilia, according to the proposal in the Chapel Hill Consensus Conference in 2012 [1]. Based on this proposal, microscopic polyangiitis (MPA) and granulomatosis with polyangiitis (GPA) also involve small-sized vessels and are associated with ANCA positivity. Our patient was diagnosed with EGPA seven years before the onset of aortic dissection, based on the following symptoms and laboratory data: bronchial asthma, rapidly progressive glomerulonephritis, peripheral neuropathy, eosinophilia, and high titers of serum MPO-ANCA.

EGPA rarely involves large vessels such as the aorta. Although there have been some cases of EGPA in which a coronary artery aneurysm developed, there has been no report of aortic involvement [3,4]. Contrarily, aortic involvement has been reported in patients with MPA and GPA [5-8]. Of note, GPA-related aortic involvement has presented as aortic wall inflammation, histologically [5]. Although the underlying mechanism is unknown, it seems likely that inflammatory and ischemic changes caused by small vessel vasculitis can injure the aortic wall, resulting in aortic aneurysm and dissection.

Conservative treatment using antihypertensive agents and steroids was sufficient to manage aortic dissection accompanied by EGPA. Type B aortic dissection can generally be managed conservatively by long-term blood pressure and imaging surveillance [2]. Most patients with aortic involvement accompanied by MPA and GPA have undergone endovascular or surgical interventions [5,7,8]; however, Skeik and colleagues advocated the effectiveness of immunosuppressants for aortitis caused by ANCA-associated vasculitis [6].

## Conclusions

EGPA can present as aortic involvement, and conservative treatment was sufficient in managing this condition. We must be aware that EGPA can cause aortic involvement, as well as cardiac involvement. Conversely, some cases of EGPA may remain unrecognized in patients with aortic aneurysm and dissection. Further reports should be accumulated to determine whether EGPA-related aortic involvement may be present much more frequently and whether conservative treatment may be sufficient to cope with this condition.

## References

[1] Jennette JC, Falk RJ, Bacon PA (2013). 2012 revised International Chapel Hill Consensus Conference nomenclature of vasculitides. Arthritis Rheum.

[2] Tadros RO, Tang GHL, Barnes HJ (2019). Optimal treatment of uncomplicated type B aortic dissection: JACC review topic of the week. J Am Coll Cardiol.

[3] Hellemans S, Dens J, Knockaert D (1997). Coronary involvement in the Churg-Strauss syndrome. Heart.

[4] Htun P, Horger M, Gawaz M, Fateh-Moghadam S (2013). Giant coronary artery aneurysms and eosinophilic granulomatosis with polyangiitis. Arthritis Rheum.

[5] Pan L, Yan JH, Gao FQ (2019). Case report of a 28-year-old man with aortic dissection and pulmonary shadow due to granulomatosis with polyangiitis. BMC Pulm Med.

[6] Skeik N, Hari G, Nasr R (2019). Aortitis caused by antineutrophil cytoplasmic antibodies (ANCA)-associated vasculitis: a case-based review. Rheumatol Int.

[7] Unlü C, Willems M, Ten Berge IJ, Legemate DA (2011). Aortitis with aneurysm formation as a rare complication of Wegener's granulomatosis. J Vasc Surg.

[8] Ryomoto M, Mitsuno M, Nishi H, Fukui S, Miyamoto Y, Hao H (2009). Aortic aneurysm due to microscopic polyangiitis. Ann Thorac Surg.

